# Scorpion Venom Active Polypeptide May Be a New External Drug of Diabetic Ulcer

**DOI:** 10.1155/2017/5161565

**Published:** 2017-10-29

**Authors:** Ting Wan, Luocheng Li, Zhanyong Zhu, Siyang Liu, Yueqiang Zhao, Mosheng Yu

**Affiliations:** ^1^Department of Plastic Surgery, Renmin Hospital of Wuhan University, Wuhan, Hubei Province 430060, China; ^2^Department of Cardiovascular Surgery, Renmin Hospital of Wuhan University, Wuhan, Hubei Province 430060, China

## Abstract

**Background:**

The epidermal growth factor (EGF) is recognized medicine of therapy in ulcer. However, its efficacy has been challenged. We compared scorpion venom active polypeptide and EGF of therapeutic effects in diabetic ulcer.

**Methods:**

The scorpion venom active polypeptide is made into gel. Fourteen diabetic SD rats were randomly divided into scorpion peptide gel group (SPG group) and EGF group. Before treatment, the rat model of diabetic ulcer was created. The levels of IL-1, IL-6, IL-8, and TNF-*α* in the wound tissue were measured at different time points during the treatment, secretions of wound were collected for bacterial culture, and the wound healing was recorded.

**Results:**

Wound healing was faster in SPG group compared to EGF group (3 weeks versus 5 weeks, *t*-test, *p* = 0.032). The levels of IL-1, IL-6, IL-8, and TNF-*α* were not statistically different when the wounds were formed but showed significant differences from the 2nd to the 5th week between two groups. The infection rate was higher in the EGF group (42.86% versus 0, Chi-square test, *p* = 0.025).

**Conclusions:**

Scorpion venom active polypeptide shortens wound healing with a stronger anti-inflammation and antibacterial effect and may be a new and effective topical drug for the treatment of diabetic ulcers.

## 1. Introduction

Diabetic foot ulcer (DFU) is one of the common and serious chronic complications of diabetes mellitus (DM) and refers to foot infections, ulcers, and/or deep tissue destruction associated with distal extremity nerve abnormalities and peripheral vascular lesions. The common consequence is chronic ulcers, the most serious outcome is amputation and even death, and the treatment cycle is long and of high medical costs, to patients with great pain and heavy burden [1]. Treating the wounds to closure as rapidly and safely as possible, therefore, is a logical strategy to reduce morbidity and resources.

There are many ways to treat diabetes ulcers, including hyperbaric oxygen therapy, autologous stem cell transplantation, debridement, negative pressure aspiration, and bioengineering skin [2–5]. But these treatments are too complex and expensive. Recently, numerous medicines are used as dressings for the treatment of chronic ulcers. Epidermal growth factor is a polypeptide containing 53 amino acid residues, which has a significant role in promoting cell division. It is currently widely used in the treatment of various wounds, ulcers, and burns [6, 7]. Unfortunately, without antibacterial effect, there is still the possibility of wound infection during the using process, to patients with additional economic losses, while increasing the workload of nursing work.

In recent years, Chinese medicine treatment of diabetic ulcer research has made great progress, and several antimicrobial peptides have been investigated as therapeutic agents [8, 9]. Scorpion venom active polypeptide (SVAP) is isolated and purified by scorpion venom. It is a biological toxin, mainly composed of nonprotein and protein, with complex physiological and pharmacological activity. Some studies have shown that scorpion venom active polypeptide has strong antilipid peroxidation and oxygen free radical elimination and has protective effect on myocardial ischemia-reperfusion injury [10–12]. In addition, several antimicrobial peptides have been found in scorpion venom, including scorpine, hadrurin, opistoporins, parabutoporin, ISCTs, StCT1, and mucroporin [13–18]. These scorpion venom peptides commonly exhibit cytolysis or microbial inhibition functions. In a previous study, researchers reported that the scorpion venom active polypeptide can rapidly destroy the cell membrane and cell wall of the bacteria, which can effectively be not only against Gram-positive bacteria but also against Gram-negative bacteria, especially for methicillin-resistant staphylococcus aureus (MRSA) and methicillin-resistant coagulase negative staphylococcus (MRCNS), and it is not easy to produce drug resistance [18, 19]; these bacteria are also common pathogens of diabetic ulcer infections. Although scorpion venom active polypeptides have so many functions, none of these studies have been validated on specific disease animal models.

The aim of this study was to evaluate the efficacy of the scorpion venom active polypeptide in the treatment of wounds in the diabetic rats.

## 2. Materials and Methods

### 2.1. Preparation of Scorpion Peptide Gel

Scorpion active venom polypeptide (SVAP) was purchased from Guangzhou snake venom institute (Guangzhou, China). For the main components and proportions of scorpion peptide gel, see Table 1.

### 2.2. Animals and Diabetic Ulcer Induction

Eight-week-old diabetic Sprague Dawley rats (130–150 g) were used in this study, and all experiments were conducted in accordance with the guidelines for the management and use of laboratory animals of China and were approved by the Institutional Ethics Committee on Animal Use (WDRY2015-K021). During treatment, rats were kept in cages with free access to water and food under conditions of controlled temperature and humidity and were subject to a 12-hour light-dark cycle. Hemorrhagic induction was used to induce ulcer in diabetic rats. The ulcer is created by clamping two-magnet disk (15 mm diameter each) on the dorsal skin of the rats, 10 mm away from the spinal column. The clamping duration (ischemic period) is 16 h, which created two 15-mm diameter ulceration wounds. During the ischemic period and for three days after clamping the rats received analgesia (Temgesic 0.5 mg/kg; Reckitt Benckiser Pharmaceuticals, Berkshire, UK). After ulcer induction in diabetic rats, they were housed individually in separate cages and randomly divided into two groups: scorpion peptide gel group (SPG group, *n* = 7) and epidermal growth factor group (EGF group, *n* = 7), respectively. In order to investigate the bacteriostasis of the drugs, a bacterial culture of wound surface secretion was performed every week.

### 2.3. Evaluation of Inflammatory Factors on Ulcer Surface

Ulcer surface secretions were collected at different time points:

1. The time of ulcer formation
2. During the treatment, in which the ulcer surface secretions were collected once a week until the ulcer surface healed

At each time point, a total of 20 *μ*l of secretions were collected from the ulcer surface. Samples were stored at −80°C and analyzed simultaneously. Levels of IL-1, IL-6, and IL-8 were detected using human ELISA Kits (IBL, Germany), whereas the TNF-*α* levels were detected using a Diagnostics TNF-*α* ELISA Kit (DRG, Germany).

### 2.4. Estimation of Ulcer Area

The ulcer area was measured immediately when the ulcer is induced, then the ulcer area was measured every three days and recorded. In the measurement, find the center of the ulcer, and then through the center point of the ulcer surface measure the length of the longest axis, and then through the center point for the vertical line of he long axis, and record the length of the vertical line. Finally, according to the shape of ulcers, different formulas were used to estimate the size of the ulcer area. Then the healing ratio was calculated by the following equation:

(1) Healing  ratio  100%=initial  wound  area−wound  areainitial  wound  area×100%.

### 2.5. Statistical Analysis

Continuous variables were presented as mean ± SD and categorical variables as number and percentage. Comparisons between the scorpion peptide antibacterial gel group and epidermal growth factor group were made using *t*-test or nonparametric equivalent Mann–Whitney test, where appropriate. Chi-square test was employed to compare categorical data between groups. *p* < 0.05 was considered statistically significant.

## 3. Results

### 3.1. Diabetic Ulcers Induction

**Figure 1:**
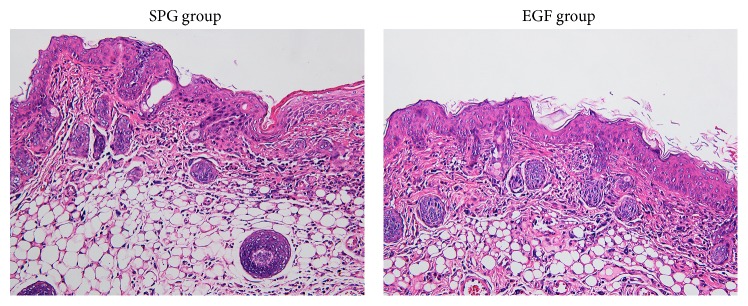
HE staining results showed a large number of neutrophils in the tissue.

All 14 diabetic rats formed ulcers. There was no death of rats during the induction. At the initial of ulcers, pathological results showed acute inflammatory response, a large number of neutrophil infiltrations (Figure 1).

### 3.2. The Antibacterial Ability of Scorpion Peptide Gel Is Better

To observe the bacterial infection on the ulcer surface, we collected ulcer surface secretions for bacterial culture before treatment, and bacterial culture of ulcer surface secretions is still performed every week after surgery. The results showed that there were no significantly differences of bacterial culture positive cases between the two groups before treatment. There was no infection situation in group A, while, in group B, there were 3 cases with bacterial infection, 2 cases occurred in the second week, and the results were positive for fecal streptococci and were sensitive to penicillin. And one rat was infected with* Staphylococcus aureus* at the third week, sensitive to cefmetazole, indicating that the incidence of infection in group B was significantly higher than that in group A (Chi-square test, *p* = 0.025) (Table 2). All 3 infected rats were given a sensitive antibiotic to clean the wound until the bacterial culture was negative.

### 3.3. The Inflammatory Factor of the Ulcer Surface Can Be Inhibited by the Scorpion Peptide Gel

**Figure 2:**
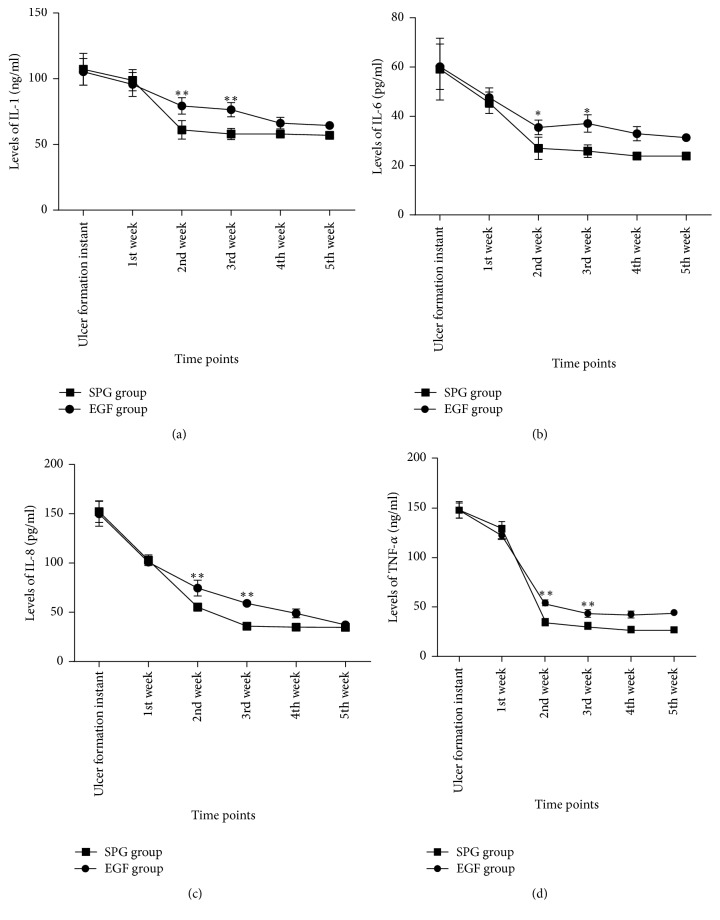
The expression of inflammatory factors of the ulcer surface has no significant differences between the two groups at the first week. From the 2nd to 5th week, the expressions of IL-1 (a), IL-6 (b), IL-8 (c), and TNF-*α* (d) were lower in SPG group obviously when compared to EGF group (*t-test, *^*∗*^*p* < 0.05 and ^*∗∗*^*p* < 0.001).

To observe the effect of two drugs on the expression of inflammatory factors on ulcer surface, we collected the secretions of ulcer surface at different time points of treatment for ELISA. The expressions of IL-1, IL-6, IL-8, and TNF-*α* in the ulcer surface secretions of all samples were increased at the early stage of ulcer formation. At the first week after induction, the ELISA results showed that the expressions of the above indicators were still higher in the ulcer surface secretions of the two groups, but there was no significant difference between the two groups. However, the expressions of inflammatory cytokines in the surface of ulcer of scorpion peptide gel group were significantly lower than that in epidermal growth factor group from 2nd week to 5th week after treatment (*t*-test, ^*∗*^*p* < 0.05 and ^*∗∗*^*p* < 0.001) (see Supplementary Tables  1–4 in the Supplementary Material available online at https://doi.org/10.1155/2017/5161565) (Figure 2).

### 3.4. Scorpion Peptide Gel Group Ulcer Healing Faster

**Figure 3:**
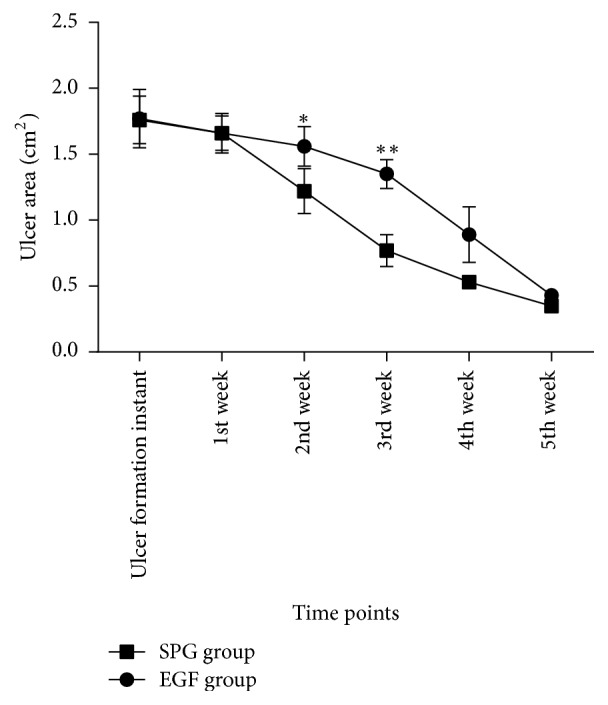
From the second week to the time of complete healing of ulcer after ulcer induction, the ulcer area of SPG group was obviously smaller than the EGF group (*t-test,*^*∗*^*p* < 0.05 and ^*∗∗*^*p* < 0.001).

**Figure 4:**
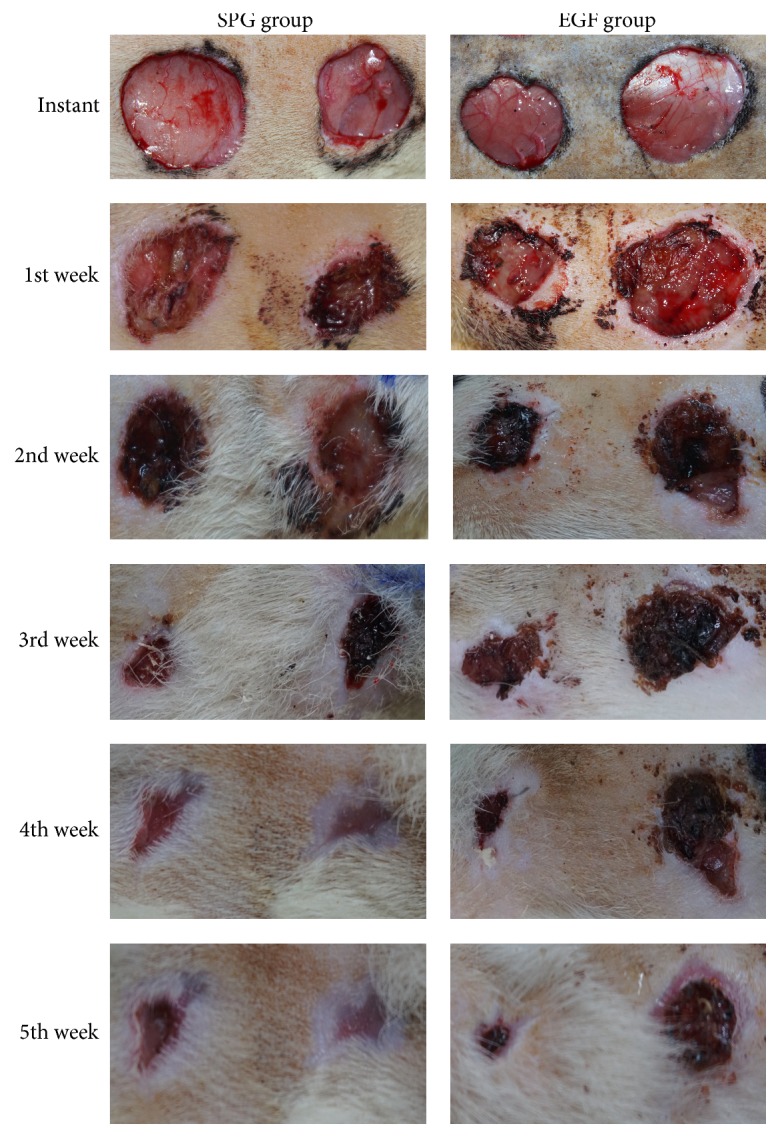
The wound healing of two groups: SPG group was significantly faster than EGF group.

The results showed that the rate of ulcer healing in rats with scorpion peptide gel group was faster than that in epidermal growth factor group. Especially from the second week to the time of complete healing of ulcer after ulcer induction, the speed of the ulcer healing has significant differences (*t*-test, ^*∗∗*^*p* < 0.001) (Supplementary Table 5) (Figures 3 and 4).

## 4. Discussion

Wound healing is a complex process of molecular biological processes, including coagulation, wound healing, inflammation, migration, and remodeling. Diabetic ulcers are one of the most common refractory chronic ulcer diseases and seriously affect the prognosis of the disease [20]. Scorpion venom active polypeptide with its anti-inflammatory, antiplasmin, antibacterial, and other pharmacological effects gradually attracted the researchers' attention. In this study, we investigated the effect for the treatment of ulcers in diabetic rats of the scorpion peptide gel compared to the epidermal growth factor.

There are many animal models currently used to study diabetic ulcers. In this study, ischemic-induced spontaneous ulcer formation was performed, the method is of high reliability, and ulcer area can be controlled, with high modulus and easy quantitative analysis. In this study, model of diabetic ulcer was created successfully by using magnetic disc compression in all rats as previously described [21]. However, the ulcers induced by this method exhibit acute inflammation; most diabetic ulcers are actually chronic inflammation; thus, an animal model that is more clinically relevant may make the data for studying diabetic ulcers more convincing.

Wound infection is an important factor in the persistence of diabetic ulcers. Therefore, the antimicrobial effect of topical drugs must be considered. In the present investigation, we performed bacterial culture of the ulcer surface secretions every week during treatment, and results showed that there were no bacterial infections at the time of ulcer formation. There were 3 cases with bacterial infection in EGF group during the treatment; two of these were infected with* Streptococcus faecalis* and one infected with* Staphylococcus aureus*; these bacteria are common infections of diabetic ulcer. Meanwhile, in the SPG group, there were no positive results for bacterial culture, indicating that the scorpion peptide gel has better anti-infective effect compared to the epidermal growth factor.

Diabetic wounds continued inflammation and repair of cell over-apoptosis is another important factor in the healing of diabetic ulcers; this is related to the excessive glucose metabolism disorder in diabetic wounds leading to overproduction of peroxides [22–24]. Several studies have reported that there were lots of inflammation factors increased in the serum and local wound of diabetic patients, including C-reactive protein, macrophage migration inhibitory factor, IL-1, IL-6, IL-8, and TNF-*α* [22, 25–27]. In the current study, we evaluated the levels of IL-1, IL-6, IL-8, and TNF-*α* of the secretions of ulcer surface at different time points during the treatment. All of the above indicators were increased at the first week after ulcer induction, but with no significant differences between two groups. From the second week to fifth week, the levels of these inflammatory indicators were significantly lower in scorpion peptide gel group when compared to the epidermal growth factor group. These results suggest that the anti-inflammatory effect of the scorpion peptide gel is better than that of the epidermal growth factor.

The most important indicator of comparison of the two-drug treatment of diabetic ulcer effect is wound healing rate. In this experiment, the rate of ulcer healing in rats in scorpion peptide gel group was faster than that in epidermal growth factor group, which obviously began from the second week after the ulcer induction. This may be due to the fact that the scorpion peptide gel can more effectively inhibit the growth of bacteria and the expression of inflammatory factors on the surface of diabetic ulcers.

We acknowledge that, in our study, animal models are acute diabetic ulcers, while there are more chronic ulcers in clinic. In addition, the mechanisms by which scorpion venom active polypeptide promotes the healing of diabetic ulcers are not further studied. More specific and in-depth cellular and/or animal experiments will strongly support this study.

## 5. Conclusions

In the present study, we found that scorpion venom active polypeptide reduces the local persistent inflammation of the wound and has better antibacterial effect in diabetic rats when compared with epidermal growth factor. The scorpion venom active polypeptide may be more suitable for the treatment of diabetic ulcers. Future clinical trials can further determine its efficacy.

## Supplementary Material

Supplementary Table 1. The expression of IL-1 on ulcer surface of two groups. The IL-1 levels were lower in SPG group from the 2^nd^ to the 5^th^ week when compared to EGF group.
Supplementary Table 2. The expression of IL-6 on ulcer surface of two groups. The IL-6 levels were lower in SPG group from the 2^nd^ to the 5^th^ week when compared to EGF group.
Supplementary Table 3. The expression of IL-8 on ulcer surface of two groups. The IL-8 levels were lower in SPG group from the 2^nd^ to the 5^th^ week when compared to EGF group.
Supplementary Table 4. The expression of TNF-α on ulcer surface of two groups. The TNF-α levels were lower in SPG group from the 2^nd^ to the 5^th^ week when compared to EGF group.
Supplementary Table 5. The ulcer area of different time points of two groups. The ulcer area of SPG group was smaller when compared to EGF group from the 2^nd^ week to the time of complete wound healing after ulcer induction.

## Figures and Tables

**Table 1:** The main components and proportions of scorpion peptide gel.

Ingredients	Proportion (%)
SVAPs	0.05
Carbomer	1
Triethanolamine	Adjust pH to 7.4
Glycerinum	10
Sodium hydrogen sulfite	0.05
Absolute ethyl alcohol	50
Azone	1
Double distilled water	Up to total volume

**Table 2:** Bacterial culture results of two groups.

		Ulcer formation	1st week	2nd week	3rd week	4th week	5th week	Total
Epidermal growth factor group	+	0	0	2	3	1	0	3
	−	7	0	5	4	6	7	
Scorpion peptide gel group	+	0	0	0	0	0	0	0
	−	7	7	7	7	7	7	
*p* value								^*∗*^0.025

+: bacterial culture positive; −: bacterial culture negative; the incidence of infection in EGF group was significantly higher than SPG group (*Chi-square test,*^*∗*^*p* = 0.025).
