# Supplementary material for: Scorpion Venom Active Polypeptide May Be a New External Drug of Diabetic Ulcer
Source: Evid Based Complement Alternat Med. 2017 Oct 29;2017:5161565. doi: 10.1155/2017/5161565 (PMC5682090; doi:10.1155/2017/5161565)
Supplement: Supplementary file 1 — Supplementary Table 1. The expression of IL-1 on ulcer surface of two groups. The IL-1 levels were lower in SPG group from the 2nd to the 5th week when compared to EGF group. Supplementary Table 2. The expression of IL-6 on ulcer surface of two groups. The IL-6 levels were lower in SPG group from the 2nd to the 5th week when compared to EGF group. Supplementary Table 3. The expression of IL-8 on ulcer surface of two groups. The IL-8 levels were lower in SPG group from the 2nd to the 5th week when compared to EGF group. Supplementary Table 4. The expression of TNF-α on ulcer surface of two groups. The TNF-α levels were lower in SPG group from the 2nd to the 5th week when compared to EGF group. Supplementary Table 5. The ulcer area of different time points of two groups. The ulcer area of SPG group was smaller when compared to EGF group from the 2nd week to the time of complete wound healing after ulcer induction. [file 5161565.f1.doc]

**Supplementary Table 1.** The expression of IL-1 on ulcer surface of two groups. The IL-1 levels were lower in SPG group from the 2^nd^ to the 5^th^ week when compared to EGF group. (*p<0.01*)

|  | Ulcer formation | 1^st^ week | 2^nd^ week | 3^rd^ week | 4^th^ week | 5^th^ week |
| --- | --- | --- | --- | --- | --- | --- |
| EGFgroup | 105.25±10.21 | 95.67±9.12 | 79.32±6.29 | 76.43±5.36 | 66.21±4.36 | 64.42 |
| n | 7 | 7 | 7 | 7 | 4 | 1 |
| SPG group | 107.22±12.04 | 98.87±8.01 | 61.11±7.03 | 57.92±4.23 | 57.88 | 56.98 |
| n | 7 | 7 | 6 | 3 | 1 | 1 |
| p-value | 0.7469 | 0.4988 | **0.0004 | **0.0008 |  |  |

**Supplementary Table 2.** The expression of IL-6 on ulcer surface of two groups. The IL-6 levels were lower in SPG group from the 2^nd^ to the 5^th^ week when compared to EGF group. (*p<0.01*)

|  | Ulcer formation | 1^st^ week | 2^nd^ week | 3^rd^ week | 4^th^ week | 5^th^ week |
| --- | --- | --- | --- | --- | --- | --- |
| EGFgroup | 60.14±9.23 | 47.65±3.94 | 35.53±2.95 | 37.09±3.53 | 32.99±2.86 | 31.34 |
| n | 7 | 7 | 7 | 7 | 4 | 1 |
| SPG group | 59.15±12.54 | 45.53±4.35 | 27.02±4.54 | 25.87±2.59 | 23.87 | 23.89 |
| n | 7 | 7 | 6 | 3 | 1 | 1 |
| p-value | 0.8692 | 0.3581 | *0.0018 | *0.0012 |  |  |

**Supplementary Table 3.** The expression of IL-8 on ulcer surface of two groups. The IL-8 levels were lower in SPG group from the 2^nd^ to the 5^th^ week when compared to EGF group. (*p<0.01*)

|  | Ulcer formation | 1^st^ week | 2^nd^ week | 3^rd^ week | 4^th^ week | 5^th^ week |
| --- | --- | --- | --- | --- | --- | --- |
| EGFgroup | 149.92±12.58 | 100.98±3.43 | 74.61±8.03 | 59.13±1.77 | 48.96±4.53 | 37.23 |
| n | 7 | 7 | 7 | 7 | 4 | 1 |
| SPG group | 152.13±11.02 | 102.92±5.28 | 55.22±4.05 | 35.99±3.03 | 34.99 | 34.76 |
| n | 7 | 7 | 6 | 3 | 1 | 1 |
| p-value | 0.7327 | 0.4308 | **0.0002 | **<0.0001 |  |  |

**Supplementary Table 4.** The expression of TNF-α on ulcer surface of two groups. The TNF-α levels were lower in SPG group from the 2^nd^ to the 5^th^ week when compared to EGF group. (*p<0.01*)

|  | Ulcer formation | 1^st^ week | 2^nd^ week | 3^rd^ week | 4^th^ week | 5^th^ week |
| --- | --- | --- | --- | --- | --- | --- |
| EGFgroup | 147.56±7.29 | 122.54±3.43 | 54.13±1.76 | 43.15±3.08 | 42.04±3.08 | 44.04 |
| n | 7 | 7 | 7 | 7 | 4 | 1 |
| SPG group | 148.35±8.36 | 129.00±7.39 | 34.22±2.63 | 30.22±1.15 | 26.71 | 26.22 |
| n | 7 | 7 | 6 | 3 | 1 | 1 |
| p-value | 0.8537 | 0.0578 | **<0.0001 | **0.0001 |  |  |

**Supplementary Table 5.** The ulcer area of different time points of two groups. The ulcer area of SPG group was smaller when compared to EGF group from the 2^nd^ week to the time of complete wound healing after ulcer induction. *(p<0.01)*

|  | Ulcer formation | 1^st^ week | 2^nd^ week | 3^rd^ week | 4^th^ week | 5^th^ week |
| --- | --- | --- | --- | --- | --- | --- |
| EGFgroup | 1.77±0.22 | 1.66±0.15 | 1.56±0.15 | 1.35±0.11 | 0.89±0.21 | 0.43 |
| n | 7 | 7 | 7 | 7 | 4 | 1 |
| SPG group | 1.76±0.18 | 1.66±0.13 | 1.22±0.17 | 0.77±0.12 | 0.53 | 0.35 |
| n | 7 | 7 | 6 | 3 | 1 | 1 |
| p-value | 0.9274 | 1 | *0.0028 | **<0.0001 |  |  |
